# Interplay Between the In-Vitro Cleaning Performance and Wear of Manual Toothbrushes

**DOI:** 10.3290/j.ohpd.b3601687

**Published:** 2022-11-23

**Authors:** Manuel J. Zoller, Florance A. Lasance, Blend Hamza, Thomas Attin, Florian J. Wegehaupt

**Affiliations:** a Resident, Clinic of Conservative and Preventive Dentistry, Center for Dental Medicine, University of Zürich, Zürich, Switzerland. Wrote the manuscript.; b Student, Center for Dental Medicine, University of Zürich, Zürich, Switzerland. Performed the experiments and wrote the German manuscript in fulfilment of requirements for a master’s degree.; c Resident, Clinic for Orthodontics and Pediatric Dentistry, Center for Dental Medicine, University of Zürich, Zürich, Switzerland. Contributed substantially to discussion, proofread the manuscript.; d Professor and Director, Clinic of Conservative and Preventive Dentistry, Center for Dental Medicine, University of Zürich, Zürich Switzerland. Research idea, contributed substantially to discussion, proofread the manuscript.; e Head, Division of Preventive Dentistry and Oral Epidemiology, Clinic of Conservative and Preventive Dentistry, Center for Dental Medicine, University of Zürich, Zürich, Switzerland. Research idea, hypothesis, experimental design, contributed substantially to discussion and writing the paper, proofread the manuscript.

**Keywords:** cleaning, performance, toothbrush, wear and tear

## Abstract

**Purpose:**

This in-vitro study deals with the question of whether the wear and tear of the manual toothbrush over a simulated timeframe up to 24 months has an effect on its cleaning performance. The purpose was to find indications as to whether and when a toothbrush needs to be replaced based on its cleaning performance.

**Materials and Methods:**

Models equipped with artificial teeth (coated with titanium dioxide) were brushed in-vitro using a brushing machine with clamped manual toothbrushes. The machine carried out even, horizontal brush strokes (120 brush strokes/min) for 1 min with a constant contact pressure of 2.5 N. The percentage of the area of titanium dioxide removed from the buccal, mesial and distal surfaces of the artificial teeth corresponded to the cleaning performance. The manual toothbrushes were used on bovine roots to simulate the wear and tear after 2, 4, 6, 12, 18, 24 months of use. The cleaning performance was re-evaluated after each simulated timepoint of wear. In addition, the brushes were photographed after each cycle.

**Results:**

An increase in the in-vitro cleaning performance of the toothbrush was observed up to 6 months of wear compared to the starting point. After that, the cleaning performance decreased somewhat, but always remained above the initial cleaning performance.

**Conclusion:**

Based on the in-vitro cleaning performance after 24 months, the toothbrush would not have to be replaced. However, this in-vitro study cannot determine when a toothbrush should be replaced, because in-vivo it is also dependent on a variety of other factors such as fraying and microbial colonisation. Direct transfer of results from this study to everyday clinical practice is therefore difficult.

In past centuries, toothpicks were used to remove annoying food residues but were not used for cleaning in the actual sense (ie, removal of oral biofilm). Due to the increasing understanding of the importance of maintaining oral hygiene by removing the oral biofilm, new methods have long since been developed. Toothbrushes can be seen as the further development of toothpicks; according to historians, the first toothbrushes were developed in China (1000 C.E.). The handles were made of ivory and the bristles of horse mane, later replaced by other animal materials, such as hog bristles set in oxbone. However, since toothbrushes were very expensive at that time, they did not become widely used. It wasn’t until the late 1930s that the bristles were made of nylon and the handles were made of plastic, making them affordable for the general populace.^11^

Some studies critically assessed the benefit of purely mechanical oral hygiene to prevent dental diseases such as caries and periodontitis.^14,33,34^ Nevertheless, mechanical plaque reduction using toothbrushes, interdental brushes and fluoridated toothpaste is still the most reliable and common method of plaque control to prevent the above mentioned dental diseases.^16,26,28,36,38,44,48^ Sufficient removal of oral biofilm depends on various factors. For example, to motivate patients to improve their own oral hygiene and thus reduce oral biofilm, they must overcome certain behavioural obstacles. This can be done, for example, by analysing the patient’s behaviour, learning and practicing suitable techniques, and strengthening them through self-observation and feedback.^20^

To mechanically remove the plaque, a very large variety of different manual and electric toothbrushes is available.^39^ Commercially available toothbrushes are labelled soft, medium and hard. These designations refer to the hardness of the bristles and are assigned by the manufacturers. For this purpose, the bristle hardness is measured using DIN ISO 22254. To do so, the brushes are guided over five rollers with a contact pressure of 5 N and the force that the bristles exert on the rollers is measured. Normalised to the bristle surface of the toothbrush, this gives the hardness in N/cm^2^. Studies showed that although hard manual toothbrushes remove statistically significantly more plaque on the buccal surfaces, their use results in more injuries to the gingiva than toothbrushes with softer bristles.^7,49^ The design of the bristles also seems to play a crucial role in removing plaque. Thus, round-ended bristles do not clean statistically significantly differently than tapered bristles, with the advantage of less gingival abrasion.^6^ There are many other features of a toothbrush that can affect cleaning performance, but which are not discussed here.

With increasing wear and tear of the toothbrush, the bristles become irreversibly bent. This is known as fraying and is the most obvious sign of wear.^45^ Studies have attempted to measure this phenomenon. For example, this includes the bending angle of the bristles^31^ or the increase in brushing surface due to bending.^13^ The study by Conforti et al^8^ tried to divide the subjective and qualitative wear of the bristles in different stages. For this purpose, the wear of the bristles was divided into 5 stages, with 0 meaning no wear and 4 meaning extreme wear.

This in-vitro study deals with the question of whether the simulated wear and tear of manual toothbrushes over time influences their cleaning performance in-vitro. The study situation is inconclusive and the transferability to the clinical situation (in-vivo) is difficult to assess. In-vivo studies on this point exist which postulate a decrease in cleaning performance over time,^13^ but there are also in-vivo studies that even show improved cleaning performance.^9^

In order to ensure sufficient plaque reduction over time, information about whether and when a toothbrush should be replaced with a new one should be provided. The null hypothesis was therefore that the service life and the associated wear and tear of a manual toothbrush has no influence on the in-vitro cleaning performance.

## Materials and Methods

### Preparation of Models and Toothbrushes

**Fig 1:**
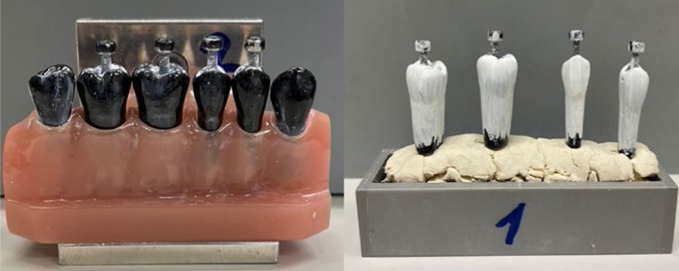
Left: tooth model with blackened teeth; right: teeth covered with titanium dioxide layer while drying.

The cleaning effect of the manual toothbrushes was tested on a model with blackened teeth (in-house production, University of Zürich, Fig 1) representing half of a mandible. The models were cast using a silicone mold based on the morphology of frasaco plastic teeth (Frasaco; Tettnang, Germany) and were made of polyurethane (Siladent; Goslar, Germany). This included the canine (tooth 13), the two premolars (teeth 14 and 15) and the three molars (teeth 16-18). In order to determine the cleaning performance planimetrically, the teeth in the model were each covered with a white layer of titanium dioxide (Fig 1). For this purpose, titanium dioxide (Merck; Darmstadt, Germany) was mixed with a 26% ethanol solution (Merck) at a mass ratio of 2:1. Teeth 14 to 17 were screwed out of the model and coated with the mixture using a brush (Kent Dental; Istanbul, Turkey). The coating with titanium dioxide was done by hand. A uniform layer thickness can therefore not be guaranteed, but the coating process was standardised as far as possible and has been used in various previous studies.^4,46^ The model teeth were left to dry for 20 min (Fig 1). Each tooth had a mesial (front of the tooth) and distal (back of the tooth) reference point. The reference points that were also covered during coating were uncovered again and the teeth were reinserted in the model. After each brushing process and the subsequent planimetric measurement, the teeth were cleaned with soap and water before coating again.

**Fig 2:**
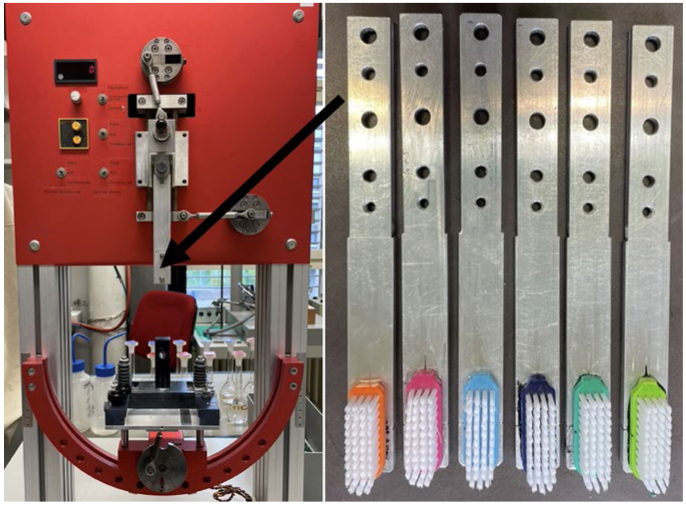
Left: ZMB2 brushing machine; right: brush heads glued on aluminum rods. The black arrow shows where the brushes were clamped.

The toothbrush used for this experiment was the Paro M43 toothbrush (Esro; Kilchberg, Switzerland). This toothbrush is listed as a reference toothbrush by the American Dental Association (ADA). It has a flat, parallel bristle field organised in 4 rows with a total of 43 filament bundles. The nylon filaments are classified as hardness-grade medium and are round-ended. A total of six Paro M43 were prepared for the brushing process. To do so, the toothbrush heads were first separated from the handle. To clamp the brush heads into the brushing machine ZMB2 (in-house production, University of Zürich, Switzerland, Fig 2), they were roughened with sandpaper (Buehler, Esslingen, Germany) and glued to clampable aluminum rods (Fig 2) with superglue (Henkel; Düsseldorf, Germany).

### Test Procedure

Two toothbrushes were assigned to each of the three tooth models and clamped as a pair in the ZMB2 machine. The toothbrush was positioned in the middle of the tooth model and contact pressure was set at 2.5N using a spring balance (Pesola; Schindellegi, Switzerland). The contact pressure of 2.5 N corresponds to the mean value of the contact pressure determined from clinical studies when brushing teeth.^12^ The machine performed a total of 60 cycles/min, ie, 120 brush strokes (back and forth). During the test, the machine brushed the model in a horizontal direction. The brushing process was carried out with the machine rotating clockwise and counterclockwise for 30 s each. In areas that contacted the toothbrush bristles, this process removed the white titanium oxide layer from the model teeth and the otherwise black tooth emerged.

**Fig 3:**
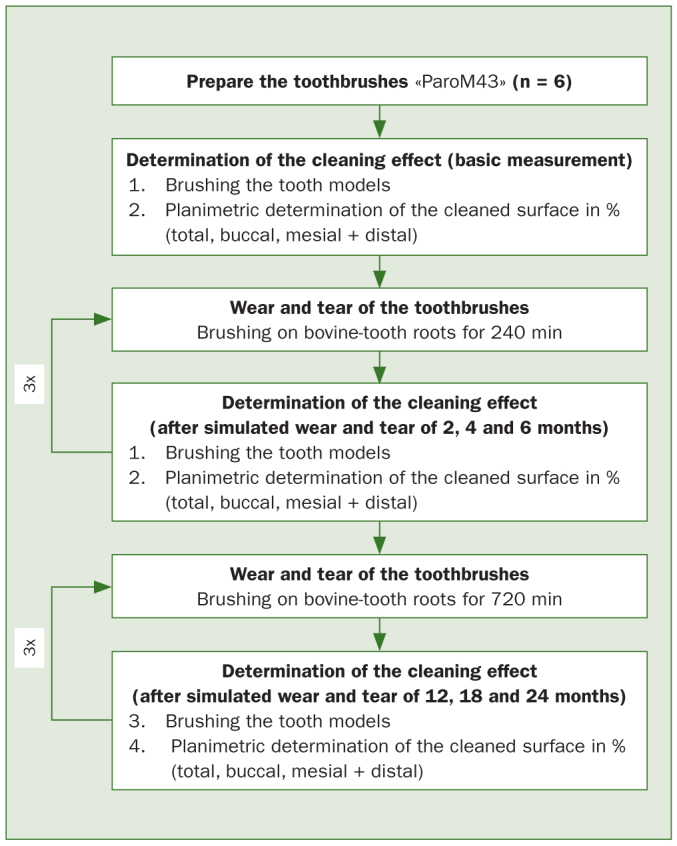
Schematic representation of the test procedure.

This was done initially with the new toothbrushes and after simulated toothbrush wear of 2, 4, 6, 12, 18 and 24 months. Figure 3 depicts a schematic representation of the test procedure.

**Fig 4:**
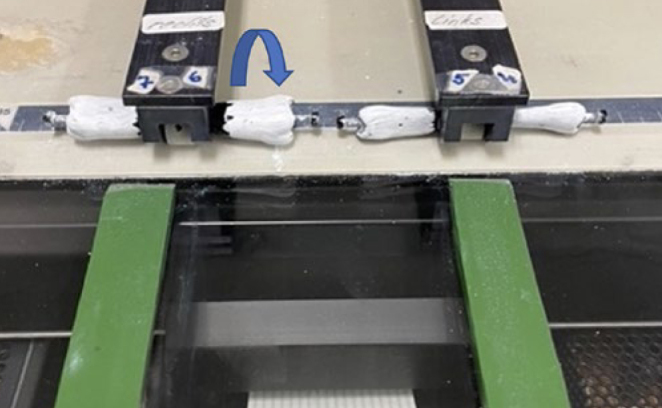
Flatbed scanner with clamped teeth. The blue arrow represents the rotation around the tooth’s axis.

### Simulated Wear and Tear of the Toothbrushes

To simulate the wear and tear of the toothbrushes through daily use, they were used on bovine roots. After reaching the desired simulated timepoints, the cleaning performance was tested again on the titanium dioxide covered models.

Two bovine tooth roots were embedded in acrylic resin (Paladur, Heraeus Kulzer; Hanau, Germany). The roots were then polished with two different Soflex disks (2382F and 1982SF, 3M Oral Care; St Paul, MN, USA) for 2 min each. A pressure of 0.4 to 0.6 N was applied to the roots and was read using a pressure gauge (Tektronix; Beaverton, OR, USA). A toothpaste slurry was prepared with two mass fractions of an Elmex caries-protection toothpaste (GABA; Therwil, Switzerland) and one mass fraction of artificially produced saliva according to Klimek et al.^22^ The toothpaste slurry was homogenised using a hand mixer (Ultra Turrax T25, IKA-Werke; Staufen, Germany) for 5 min.

The toothbrushes were artificially worn down using the ZMB8 brushing machine. To do so, the toothbrushes, the embedded bovine roots and a container with 72 g of the toothpaste slurry were clamped into the machine. A bovine tooth sample was assigned to each toothbrush and was not exchanged. The contact pressure of the toothbrush was set to 2.5 N and checked using a spring balance. The toothbrushes scrubbed vertically over the bovine roots. The speed of the machine was set at 60 cycles/min, which means that during one minute, 60 forward and 60 backward movements took place over the bovine roots. After each wear cycle, the cleaning performance of the toothbrushes was determined again, as described above. The first three wear cycles consisted of 240 min each and cycles 4 to 6 consisted of 720 min each. The total number at the various points in time (after 1, 3, 6, 12, 18 and 24 months) was calculated by multiplying the number of brush strokes per month (28800) by the respective number of months. This means that after 24 months of simulated wear, a total of 691,200 brush strokes were performed. The number of brush strokes used corresponds to the usual number under clinical conditions according to Wiegand and Attin.^47^

### Planimetric Determination of the Cleaned Areas

**Fig 5:**
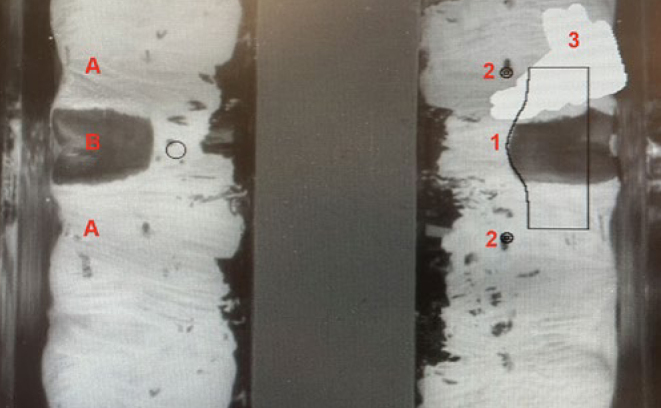
Scan with visible mask. A = approximal surfaces; B = buccal surfaces; 1 = superimposed mask; 2 = reference points; 3 = areas manually painted white.

In order to determine the proportion of surfaces cleansed of titanium dioxide, the four brushed teeth (17 to 14) were clamped into a flatbed scanner (Hewlett-Packard; Palo Alto, CA, USA) modified with an unrolling device (in-house production, University of Zürich, Switzerland, Fig 5). The teeth rotated once around their own axes in the device for the scan. The program DeskScan II (Hewlett-Packard) was used for the scan. The settings were as follows: type = black and white photo, brightness and contrast = 125%, and scaling = 300%. Care was taken to ensure that the reference points were clearly visible on the respective scan, so that the program could superimpose a mask over the corresponding scan.

The scans were evaluated with the adapted program Image 1.61 FAT Bracetts (in-house production, University of Zürich, Switzerland). Interdentally, the scanner usually recognises the white areas as too dark, which is why they were painted over white manually in the program. Small damaged areas on the model’s blackened teeth were corrected using gray tones. With the help of the program, the percentage of cleaned total, buccal, mesial and distal areas (black spots) compared to the total area of the respective tooth surfaces were determined. For better understanding, the evaluation with the mask is shown in Fig 5.

### Statistical Analysis

The statistical evaluation was carried out using Microsoft Excel (Microsoft; Redmond, WA, USA) and the development environment R for the programming language R (R Core Team, https://www.R-projec.org) using the packages Ggplot 2 (Wickham H., https://ggplot2.tidyverse.org), ImerTest (Kuznetsova et al., https://github.com/runehaubo/ImerTestR) and multcomp (Hothorn et al., https://multcomp.r-forge.r-project.org).

A linear mixed model was calculated for each of the four different surfaces (total, buccal, mesial, and distal). The cleaned area was explained by the variable measuring time (after 2, 4, 6, 12, 18, 24 months). In addition, the variable “tooth model” was included as a random effect to account for the dependency of the observations of the same toothbrush. Using ANOVA, the null hypothesis “there is no difference in the cleaned surfaces between the measurement times” was tested. If p < 0.05, the null hypothesis was rejected. Then the different measurement times were compared with the initial time (0 months) in order to test the null hypothesis: there is no difference in the cleaned area between measurement time 0 and measurement time x. Here, too, the significance level was set at 5% (p < 0.05). The p-values were additionally corrected per area for multiple testing using Dunnett’s test.

## Results

The ANOVAs for the “total” and “buccal” areas showed statistically significant changes (p < 0.05) in the area of the cleaned surface between the different measurement times. In contrast, the “mesial” and “distal” areas showed no statistically significant differences (p > 0.05) between the measurement times within one area. The values determined per area and the statistical significance compared to the time “0 months” are shown in Table 1.

**Table 1:** Mean values ± SD of the cleaned surface in % after different durations of wear and tear of the toothbrushes for the different investigated areas

		Duration [months]						
		0	2	4	6	12	18	24
Area [% cleaned surface ± standard deviation]	Total	39.1 ± 1.5	42.5 ± 1.9	43.5 ± 2.1	44.8 ± 1.3	42.3 ± 2.2	41.9 ± 1.3	41.3 ± 2.0
	Significance	A	B	B	B	B	B	B
	Buccal	71.9 ± 4.4	77.2 ± 3.5	79.6 ± 3.7	81.7 ± 2.1	77.5 ± 3.2	76.9 ± 3.4	76.1 ± 3.7
	Significance	A	B	B	B	B	B	B
	Mesial	2.9 ± 3.1	5.1 ± 6.5	3.9 ± 4.1	3.2 ± 3.9	3.7 ± 4.7	3.8 ± 4.6	3.0 ± 4.0
	Significance	A	B	A	A	A	A	A
	Distal	0.2 ± 0.2	0.4 ± 0.2	0.6 ± 0.4	0.8 ± 0.8	0.3 ± 0.3	0.3 ± 0.3	0.4 ± 0.3
	Significance	A	A	A	A	A	A	A

The statistical significance is read exclusively in the horizontal direction and is always only compared with the time “0 months” (baseline). Values with the capital letter B differ statistically significantly from the start point “0 months” (p < 0.05), values with the capital letter A do not differ from timepoint “0 months” (p > 0.05).

For the total surface, the cleaned surface area (mean ± SD) after 2 (42.5% ± 1.9), 4 (43.5% ± 2.1), 6 (44.8% ± 1.3), 12 (42.3% ± 2.2), 18 (41.9% ± 1.3) and 24 months (41.3% ± 2.0) was statistically significantly greater than at 0 months (39.1 ± 1.5) (p < 0.05). The same applies to the buccal surface, the cleaned surface area (mean ± standard deviations) after 2 (77.2% ± 3.5), 4 (79.6% ± 3.7), 6 (81.7% ± 2.1), 12 (77.5% ± 3.2), 18 (76.9% ± 3.4), 24 (76.1% ± 3.7) was statistically significantly higher than at 0 months (71.9% ± 4.4) (p < 0.05).

For the mesial surface, only the cleaned surface area (mean ± SD deviations) after 2 months (5.1% ± 6.5) was significantly greater than at 0 months (2.9% ± 3.1), (p < 0.05, respectively). However, after 4 (3.9% ± 4.1), 6 (3.2% ± 3.9), 12 (3.7% ± 4.7), 18 (3.8% ± 4.6) and 24 months (3.0% ± 4.0) no statistically significant difference could be determined compared to the time point 0 months (p > 0.05).

The cleaned distal surface area (mean ± SD) after 2 (0.4% ± 0.2), 4 (0.6% ± 0.4), 6 (0.8% ± 0.8), 12 (0.3% ± 0.3), 18 (0.3% ± 0.3), and 24 months (0.4% ± 0.3) was not significantly larger than at 0 month (0.2% ± 0.2), (p > 0.05, respectively).

**Fig 6:**
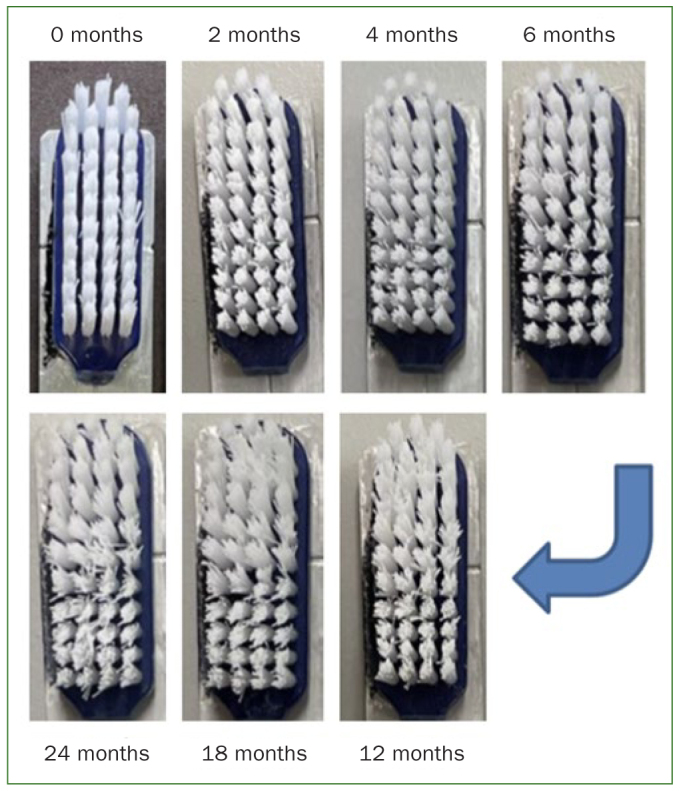
Images of a toothbrush head at different times of simulated in-vitro wear.

Figure 6 shows the appearance of a toothbrush head at different points in time. At time 0 months, the bristles were new and had a wear index of 0 according to Conforti et al.^8^ After 2 and 4 months of simulated wear, the bristles were assigned a wear index of 1, as the outer bristles began to splay. With increasing wear, the inner bristles also started to splay. After 6 months, a wear index of 2 was reached. After 12, 18 and 24 months. a wear index of 3 was found. as both the outer and inner bristles showed visible splaying. A wear index of 4 – where no distinction between the inner and the outer tufts could be made – was not achieved at any time.

## Discussion

Counterintuitively, the present results showed that the in-vitro cleaning performance increased as the toothbrush wore down compared to the unused/new toothbrush. At all observation times (after 2, 4, 6, 12, 18 and 24 months), a statistically significantly higher cleaning performance was determined for the total area compared to the time point 0 months. The null hypothesis that there is no difference in the in-vitro cleaning performance between the different observation times must therefore be rejected. It was observed that the in-vitro cleaning performance increased continuously from 0 to 6 months, when it reached its peak. The in-vitro cleaning performance then decreased again up to 24 months, but always remained above the initial cleaning performance. A possible explanation for this phenomenon is that that the bristles became shorter during the experiment due to wear. However, since the diameter of the filaments remain the same, this could lead to an initial increase in rigidity and thus cleaning performance. With increasing wear, the splaying effect plays a role play, where the bristles bend irreversibly. This enables the bristles to cling better to the surface of the teeth and thus increase the mechanical contact area. The decrease after 6 months could be related to the advanced fraying of the bristles, so that the contact pressure of 2.5 N is no longer sufficient to bring the bristles into sufficient contact with the tooth.^43^ The study by Van Leeuwen et al^45^ showed that the in-vivo plaque index in the participants correlated weakly positively with increasing wear score (according to Conforti et al^8^). This means that the cleaning performance decreased while the wear score increased, which was particularly the case with a wear score of 4. However, caution is advised, as other factors also seem to play an important role in cleaning performance. The study by Kaiser et al^21^ showed that two electric brush heads with no statistically significant differences in wear score (according to Rawls et al^32^) after one month of use had a statistically significantly different cleaning performance. The only difference between those two electric brush heads was that the brush head with a better cleaning performance had 12% more filaments. This situation also suggests the influence of further factors.

Another important point is that the majority of the cleaned surfaces were buccal, since the mesial and distal cleaning in-vitro was generally poor. Here the in-vitro cleaning performance increased for the mesial area at 2 months, but thereafter showed no statistically significant differences vs 0 months. No significant differences at all could were found for the distal surface compared to 0 months. In summary, in this study, statistically significant differences at all the timepoints of wear were only found for the buccal surfaces.

In comparison to other studies, there is no consensus regarding the cleaning performance of toothbrushes used over time in-vivo. Some studies showed a statistically significant decrease in cleaning performance with increasing service time in-vivo.^1,13^ In contrast, other in-vivo studies did not observe any statistically significant differences,^17,41,42^ with one study even postulating an increase in cleaning performance in-vivo.^9^ However, it should be noted that the comparability of this study with others, especially in-vivo studies, is limited. The in-vitro tests are idealised, with the same contact pressure and standardised brushing movements. Additionally, many other factors influence the results, such as different test arrangements, toothbrushes, surfaces, scores, and wear times, which makes it difficult to compare the different studies.

Paro M43 toothbrushes have been used in many other studies, consisting mostly of cleaning or abrasion tests.^35,40,50^ Paro M43 toothbrushes are also used for the highly standardised process of determining RDA and REA values of toothpastes.^15^ However, there are numerous other studies that used a different type of toothbrush.^25,41,42^ It is clear that a single toothbrush cannot be representative of all toothbrushes used in daily life. Thus, the results of this in-vitro study only apply to toothbrushes with a plane brush-head design, such as the Paro M43, which does not correspond to the majority of toothbrushes available on the market.

In this experiment, the counterpart to the toothbrush was the titanium oxide-ethanol mixture, which acted as a plaque substitute. It is clear that this does not fully reflect the physical properties of dental plaque. These include viscosity, water insolubility, and high abrasion resistance, among others. However, there is no standardised procedure for producing artificial plaque. The study by Imfeld et al^19^ describes the titanium dioxide-ethanol mixture also used in this study. The titanium dioxide ethanol coating does not flake off and is therefore only removed from those areas that are effectively touched by the filaments of the toothbrush. Therefore, the surfaces freed from the coating can be considered as cleaned. The use of toothpaste was deliberately avoided, as the coating is water soluble and would therefore simply dissolve.

In many clinical toothbrushing studies, subjects used the Bass technique or the modified Bass technique.^35,41,42,45^ The horizontal brushing motions used in this study are clinically associated with an increased incidence of periodontal and wedge-shaped defects;^5,19,27,30,37^ in spite of this, a large proportion of the population still uses the horizontal scrubbing method.^19^ In the future, however, further studies with different brushing techniques would be useful.

According to Wiegand et al,^47^ the usual number under clinical conditions is 10 to 15 brush strokes per tooth. For an adult with all 28 teeth and 10 to 15 brush strokes per tooth, 280 to 420 brushing strokes can be expected over a cleaning period of 2 to 3 min. The 120 brush strokes per minute used here are therefore within this range of toothbrush wear. In addition, some studies have shown that longer brushing times are not necessarily associated with an increased plaque reduction, because patients simply repeatedly skip the same areas.^2,18^ It must also be borne in mind that many aspects of toothbrushing are not clear in the literature, such as frequency and time point (before or after meals).^3^

The present study examined the in-vitro cleaning performance of a manual toothbrush over a time period of 24 months. Assuming that a large part of the population brushes their teeth at least twice a day for two minutes each time, this results in a usage time of around 48 h within the 24 months. For this reason, the toothbrushes were worn with 120 brush strokes per minute on the bovine roots until each of the six desired wear points were reached. Of course, the wear of the filaments depends on many other factors not considered here, such as the users themselves, the respective habits, anatomy of the oral cavity, brushing duration, brushing frequency, brushing technique and contact pressure.^10,23,24,29^ Even the use of different toothpastes can have an impact on the morphology of the toothbrush bristle tips.^32^ For this reason, a toothpaste slurry was also used for the respective wear processes to allow including this influence on the bristles of the toothbrushes. However, idealised artificial wear does not directly reflect the wear that can occur from a person’s individual toothbrushing habits. Moreover, the saliva, oral microorganisms, interactions with food debris, and the natural aging of nylon bristles over time may have an impact in-vivo. On the other hand, in these idealised in-vitro experiments, it was possible to exclude interfering factors that can occur in a clinical study, thus enhancing comparability between the different timepoints measured.

In general, a toothbrush replacement is recommended by the ADA after 3 months of use. This means after a total of 365 min of use within these 3 months, which corresponds to 43,800 brush strokes used in the present study. However, as already mentioned, there is disagreement in the literature. In addition, most studies only examined a period of 3 to 6 months. This study therefore aimed to examine a much longer period of up to 24 months. The results of this study challenge the ADA recommendation to change toothbrushes every 3 months.^35^ However, based on the available results of this in-vitro experiment, it cannot be concluded that no toothbrush replacement is necessary for 24 months. This in-vitro study gives an indication of the mechanical cleaning performance of worn bristles, but not when a toothbrush should be replaced. Other factors play an important role and should be taken into when replacing the toothbrush: in particular, fraying, microbial colonisation of the toothbrush, and the risk of injury to the gingiva with increasing wear.

This in-vitro study was unable to conclusively determine the optimal timepoint in terms of cleaning performance at which a toothbrush should be replaced. Nevertheless, the lifespan of a toothbrush in-vivo may depend more on its appearance than on how long it is used. In the future, a comparison of toothbrushes with different bristle hardnesses and geometry with regard to their cleaning performance after wear could definitely be useful. The examination of other brushing parameters, such as the brushing technique, is also conceivable.
